# Tea consumption and cardiovascular risk and outcomes: mechanistic insights, epidemiological evidence, and clinical implications

**DOI:** 10.3389/fcvm.2026.1913976

**Published:** 2026-09-03

**Authors:** Rong Liu, Jialin Li, Yanchun Liang

**Affiliations:** 1 Department of Cardiology, General Hospital of Northern Theater Command, Shenyang, China; 2 Department of Cardiology, Beijing Anzhen Hospital, Capital Medical University, Beijing, China

**Keywords:** bioactive compounds, cardiovascular disease, prognosis, tea consumption, translation

## Abstract

Cardiovascular disease (CVD) remains a leading cause of morbidity and mortality worldwide. Tea contains bioactive compounds with plausible antioxidant, anti-inflammatory, endothelial, metabolic, antithrombotic, and gut microbiota-mediated effects. Observational studies generally associate habitual tea consumption with lower risks of atherosclerotic CVD and mortality, but causal inference remains uncertain because secondary-prevention data are limited, randomized trials focus mainly on surrogate outcomes, and Mendelian randomization has not consistently supported causal cardiovascular effects. Differences in tea composition and patient characteristics provide a rationale for individualized tea-consumption strategies; however, current evidence does not support disease-specific tea selection or a standardized therapeutic dose. Interpretation is further limited by residual confounding, reverse causation, heterogeneous exposure definitions, bioavailability, and safety considerations. Future studies should standardize tea exposure and evaluate clinical cardiovascular endpoints in adequately powered randomized trials to determine whether personalized tea strategies have clinical value.

## 1 Introduction

Cardiovascular disease (CVD) remains a leading cause of morbidity and mortality worldwide (1). Tea, derived from Camellia sinensis, is widely consumed and contains diverse bioactive constituents, including catechins, theaflavins, thearubigins, and theabrownins, that may influence pathways involved in oxidative stress, inflammation, endothelial dysfunction, thrombosis, and cardiometabolic regulation (2). Experimental studies provide substantial biological plausibility, while epidemiological studies have frequently associated habitual tea consumption with favorable cardiovascular outcomes. However, these findings arise from heterogeneous evidence streams—including preclinical experiments, trials of surrogate risk markers, observational cohorts, and genetic analyses—and therefore differ substantially in their ability to support causal inference.

**Figure 1:**
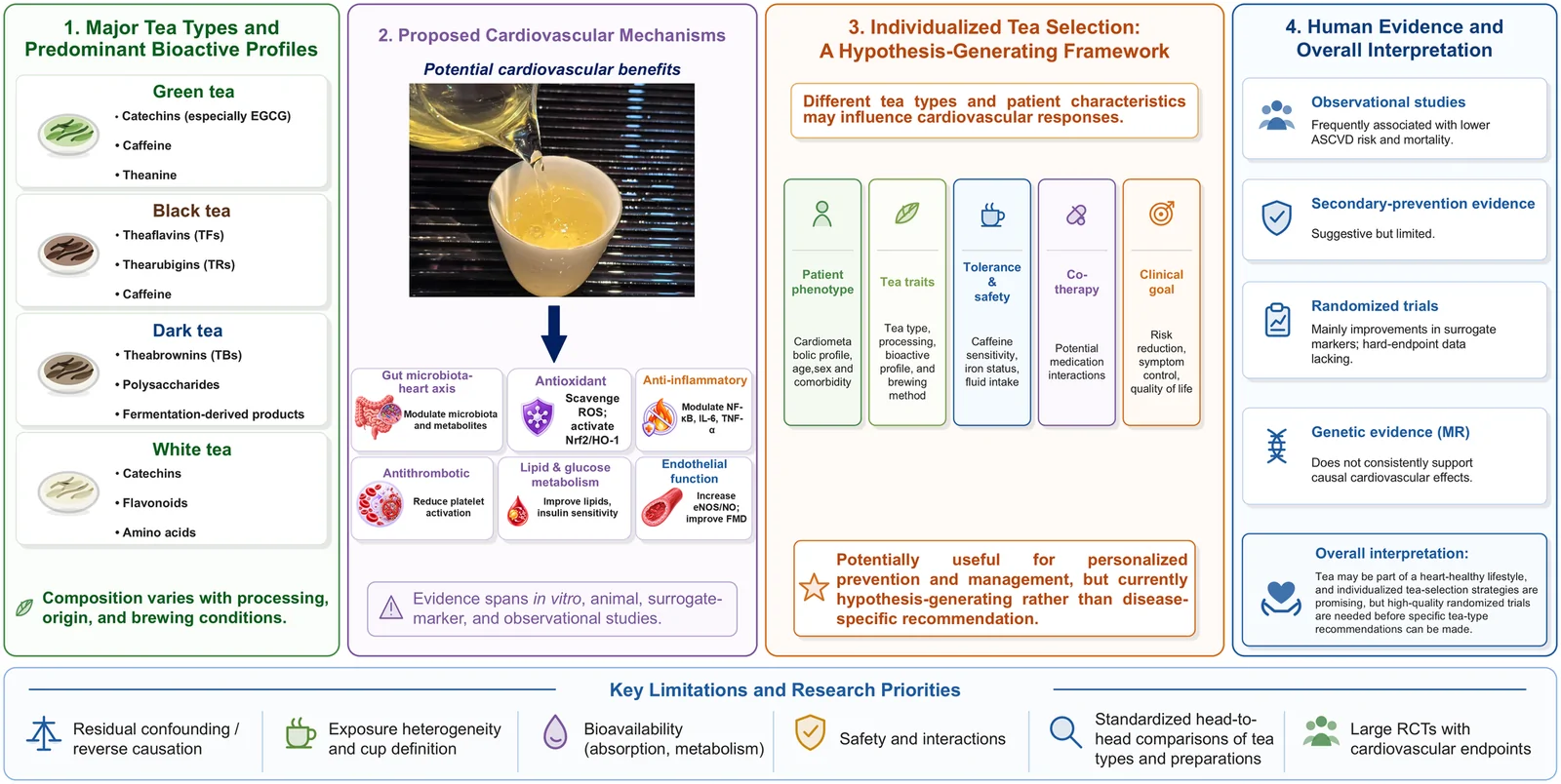
Mechanistic rationale, clinical evidence, and a hypothesis-generating framework for individualized tea consumption strategies in cardiovascular health. Tea types differ in their predominant bioactive constituents, including catechins, theaflavins, thearubigins, and theabrownins (Panel 1). These compounds may influence cardiovascular biology through multiple pathways, including oxidative stress, inflammation, endothelial function, metabolism, thrombosis, and the gut microbiota–heart axis (Panel 2). Differences in tea composition and patient characteristics provide a rationale for individualized tea-consumption strategies; however, such approaches remain hypothesis-generating rather than disease-specific recommendations (Panel 3). Current human evidence includes observational studies, randomized trials of surrogate outcomes, and genetic analyses, with persistent limitations related to causality, exposure heterogeneity, bioavailability, and safety (Panel 4). Future studies are required to determine whether personalized tea strategies translate into clinically meaningful cardiovascular benefits. ASCVD, atherosclerotic cardiovascular disease; EGCG, epigallocatechin-3-gallate; FMD, flow-mediated dilation; MR, Mendelian randomization; ROS, reactive oxygen species; TFs, theaflavins; TRs, thearubigins; TBs, theabrownins.

Variation in tea processing and bioactive composition further raises the possibility that cardiovascular responses may differ among tea types and patient characteristics. Such heterogeneity provides a rationale for exploring individualized tea-consumption strategies, but current evidence is insufficient to support disease-specific tea selection or therapeutic dosing. This narrative Mini Review critically integrates mechanistic, epidemiological, clinical, and safety evidence on tea consumption and cardiovascular risk and outcomes, with particular attention to causal limitations, bioavailability, exposure heterogeneity, and the potential role of individualized tea selection as a hypothesis-generating framework for future clinical research (Figure 1).

## 2 Literature search strategy

PubMed was searched from database inception through July 2026 using terms related to tea and its major constituents (including green, black, and dark tea, EGCG, theaflavins, thearubigins, and theabrownins) combined with cardiovascular outcomes, risk factors, mechanisms, bioavailability, and safety. Reference lists of relevant reviews and key studies were also screened. Peer-reviewed studies evaluating Camellia sinensis tea or defined tea constituents in relation to cardiovascular outcomes, cardiometabolic risk factors, mechanisms, dose-response relationships, or safety were considered. Non-tea herbal infusions, duplicate reports, and studies without cardiovascular relevance were excluded. Human evidence was prioritized for clinical associations and safety, while representative experimental studies were retained for mechanistic context. Both supportive and discordant findings were considered. Given the narrative scope of this mini Review, no formal risk-of-bias assessment or *de novo* quantitative synthesis was performed. The literature search was limited to PubMed and was intended to identify representative evidence across mechanistic, epidemiological, and clinical domains rather than to constitute a systematic, exhaustive literature search.

## 3 Bioactive constituents and mechanistic evidence

Tea contains catechins, especially EGCG in minimally fermented green tea, together with oxidation products such as theaflavins (TFs) and thearubigins (TRs) in black tea and fermentation-related theabrownins (TBs) in dark tea (2). These compounds have been studied across multiple experimental systems, but the evidence level varies substantially.

### 3.1 Oxidative stress, inflammation, and endothelial function

Oxidative stress and chronic inflammation contribute to atherosclerosis and adverse cardiovascular remodeling (3). In cell and animal models, EGCG can scavenge reactive oxygen species, activate Nrf2/HO-1 signaling, inhibit NADPH oxidase and LOX-1-related pathways, and suppress NF-kB-mediated inflammatory signaling (4, 5). TFs have also shown antioxidant and anti-inflammatory activity in experimental models. These findings provide biological plausibility but do not establish that concentrations achieved after ordinary tea drinking reproduce the effects observed *in vitro*.

Endothelial function represents one human bridge between mechanism and clinical phenotype. Experimental work indicates that EGCG can activate endothelial nitric oxide synthase (eNOS) (6), while a meta-analysis of nine intervention studies found that tea consumption increased flow-mediated dilation (FMD) by 2.6 percentage points (95% CI 1.8–3.3) (7). FMD itself is associated with future cardiovascular risk; a meta-analysis of 23 prospective studies reported a pooled CVD risk of 0.92 (95% CI 0.88–0.95) per 1% higher FMD (8). Nevertheless, FMD remains a surrogate marker, and improvement in FMD cannot be assumed to translate into fewer clinical events.

### 3.2 Cardiometabolic, antithrombotic, and myocardial effects

Tea constituents may influence blood pressure, lipids, glucose metabolism, and platelet function. A meta-analysis of 13 randomized trials involving 1,367 participants reported modest reductions in systolic blood pressure (−1.98 mmHg, 95% CI −2.94 to −1.01) and diastolic blood pressure (−1.92 mmHg, 95% CI −3.17 to −0.68) with green tea (9). Experimental studies suggest that EGCG and TFs can reduce platelet activation through several pathways (10, 11). Joo et al. further showed *ex vivo* modulation of platelet aggregation when EGCG was added to blood obtained from healthy volunteers receiving antiplatelet agents (12). These findings are hypothesis-generating; they do not establish clinical bleeding safety or benefit after percutaneous coronary intervention.

Preclinical studies also suggest anti-apoptotic and antifibrotic effects of EGCG and protective effects of TF derivatives in myocardial ischemia/reperfusion models (13, 14). Evidence from non-cardiac organ ischemia/reperfusion models was not used to support myocardial protection in this revision.

### 3.3 Gut microbiota-heart axis

Tea-microbiota interactions are an emerging mechanism. Gut-derived metabolites, including trimethylamine N-oxide, have been associated with major adverse cardiovascular events in prospective studies (15). In preclinical models, TB-rich dark-tea products can alter microbial composition, bile-acid metabolism, and short-chain-fatty-acid production (16, 17). Such findings are mechanistically relevant, but most evidence for specific TB-microbiota pathways remains preclinical, and translation to long-term cardiovascular outcomes in humans has not been demonstrated.

### 3.4 Bioavailability and translational limitations

Bioavailability is a major constraint on mechanistic interpretation. In a human pharmacokinetic study, the peak plasma EGCG concentration after a green-tea preparation was 77.9 ± 22.2 ng/mL, approximately 0.17 µmol/L, with substantial interindividual variability (18). This illustrates that systemic exposure after beverage-scale intake can be far below concentrations commonly used in cell experiments. Larger and chemically heterogeneous oxidation or polymerization products, including TRs and TBs, have less well-characterized direct systemic exposure, and colonic microbial transformation may contribute importantly to their biological effects (17). Accordingly, evidence from purified compounds, rodent dosing, concentrated extracts, and brewed-beverage consumption should be considered distinct rather than pooled into a single mechanistic-to-clinical continuum.

## 4 Epidemiological and clinical evidence

Representative human studies are summarized in Table 1, with study design, exposure definition, effect estimates, adjustment approach, and major limitations shown to make the evidence hierarchy explicit.

**Table 1:** Representative human evidence on tea consumption and cardiovascular risk or outcomes.

Study	Design/population	Exposure	Outcome/effect estimate	Follow-up	Main adjustment approach	Key interpretation/limitation
Ras et al. (7)	Meta-analysis; 9 intervention studies	Tea beverage/intervention	FMD +2.6 percentage points (95% CI 1.8–3.3)	Intervention studies	Study-level meta-analysis	Human surrogate endpoint; does not establish event reduction.
Peng et al. (9)	Meta-analysis; 13 RCTs, *n* = 1,367	Green tea	SBP −1.98 mmHg (95% CI −2.94 to −1.01); DBP −1.92 mmHg (−3.17 to −0.68)	Trial durations varied	Randomized comparisons pooled	Modest risk-factor effect; not a hard cardiovascular endpoint.
China-PAR (19)	Prospective cohort; China, *n* = 100,902	Habitual tea >=3 times/week vs. never/non-habitual	Incident ASCVD HR 0.80 (0.75–0.87); ASCVD mortality HR 0.78 (0.69–0.88); all-cause mortality HR 0.85 (0.79–0.90)	Median 7.3 y	Multivariable Cox models including demographic, lifestyle and clinical factors	Primary prevention; self-reported exposure; residual confounding remains possible.
UK Biobank (20)	Prospective cohort; UK, *n* = 498,043	Tea cups/day; black tea predominant	2–3 cups/day vs. none: all-cause mortality HR 0.87 (0.84–0.91)	Median 11.2 y	Age, sex, ethnicity, deprivation, health status, BMI, smoking, activity, alcohol, coffee and dietary factors	Large non-Asian cohort; observational and self-reported exposure.
JACC (21)	Prospective cohort; Japan; MI survivors *n* = 1,214	Green tea: none to >=7 cups/day	MI survivors, >=7 cups/day vs. none: all-cause mortality HR 0.47 (0.30–0.72); deaths 40 vs. 59	Median 18.5 y	Multivariable Cox models for demographic, lifestyle, dietary and clinical factors	Secondary-prevention association; very high intake category; nonrandomized exposure.
Liu et al. (22)	Case-control; China, *n* = 801 (401 AF, 400 controls)	Green-tea use; frequency, concentration, and duration	Green-tea intake: adjusted OR for AF 0.349 (0.253–0.483)	Not applicable	Age, sex, BMI, smoking, alcohol, physical activity, hypertension, hyperlipidemia, diabetes and CAD	Large apparent effect; vulnerable to recall, selection and residual confounding.
Cai et al. (23)	Two-sample Mendelian randomization	Genetically proxied tea consumption	No causal association with AF or six other cardiovascular disorders	Genetic causal inference	Instrumental-variable analyses plus sensitivity tests	Does not support a causal interpretation; depends on instrument validity and exposure proxy.
Yang et al. (27)	Cross-sectional CNHS 2015–2017; China, *n* = 43,757	>5 cups/day vs. non-habitual	MetS OR 0.836 (0.771–0.905); central obesity OR 1.354 (1.236–1.484)	Cross-sectional	Multiple-adjusted logistic regression	Prevalence, not incidence; opposing associations across MetS components.
Yu et al. (28)	Prospective rural cohort; China, *n* = 3,632 without baseline MetS	Occasional and daily tea-drinking categories	Incident MetS: occasional OR 1.284 (1.050–1.570); 1–2 times/day OR 1.376 (1.030–1.760)	Median 4.66 y	Multivariable regression for demographic and lifestyle factors	Discordant prospective evidence; exposure pattern and regional context may limit generalizability.

AF, atrial fibrillation; ASCVD, atherosclerotic cardiovascular disease; BMI, body mass index; CAD, coronary artery disease; CI, confidence interval; CNHS, China Nutrition and Health Surveillance; DBP, diastolic blood pressure; FMD, flow-mediated dilation; HR, hazard ratio; MI, myocardial infarction; MetS, metabolic syndrome; OR, odds ratio; RCT, randomized controlled trial; SBP, systolic blood pressure.

### 4.1 Atherosclerotic cardiovascular disease

In the China-PAR project, 100,902 adults without established ASCVD were followed for a median of 7.3 years. Habitual tea consumption (three or more times per week) was associated with lower risks of incident ASCVD (HR 0.80, 95% CI 0.75–0.87), ASCVD mortality (HR 0.78, 95% CI 0.69–0.88), and all-cause mortality (HR 0.85, 95% CI 0.79–0.90) (19). These are primary-prevention associations rather than prognosis among patients with coronary heart disease. Evidence from a non-Asian population is provided by the UK Biobank. Among 498,043 participants followed for a median of 11.2 years, in a setting where black tea predominated, tea intake was associated with lower all-cause mortality across several intake categories; for example, 2–3 cups/day versus none was associated with HR 0.87 (95% CI 0.84–0.91) (20). Extensive multivariable adjustment was performed, but residual confounding remains possible. Secondary-prevention evidence is more limited. In the Japan Collaborative Cohort Study, 1,214 myocardial infarction (MI) survivors were categorized from no green-tea intake to at least seven cups/day and followed within a larger cohort for a median of 18.5 years. Among MI survivors, the multivariable HR for all-cause mortality was 0.47 (95% CI 0.30–0.72) for at least seven cups/day versus none; 40 deaths occurred in the highest-intake group and 59 in the reference group (21). The result is notable but remains based on self-reported, nonrandomized exposure.

### 4.2 Arrhythmias

A Chinese case-control study of 801 participants reported a strong inverse association between green-tea intake and atrial fibrillation (AF) (adjusted OR 0.349, 95% CI 0.253–0.483) (22). The magnitude of this estimate warrants particular caution because case-control exposure assessment, residual confounding, and healthy-user behavior can generate large associations. A two-sample Mendelian randomization study subsequently found no evidence that genetically proxied tea consumption causally affected AF or six other cardiovascular disorders (23). Therefore, current data do not justify using tea to prevent AF or recurrence after ablation.

### 4.3 Metabolic syndrome and cardiometabolic risk

Metabolic syndrome (MetS) is associated with increased cardiovascular risk (24). Randomized-trial meta-analyses suggest small favorable changes in several MetS components (25, 26), but these are risk-factor outcomes rather than cardiovascular events. Observational findings are inconsistent. In the cross-sectional China Nutrition and Health Surveillance 2015–2017 analysis of 43,757 adults, intake above five cups/day was associated with lower odds of prevalent MetS (OR 0.836, 95% CI 0.771–0.905) but higher odds of central obesity (OR 1.354, 95% CI 1.236–1.484) (27). Conversely, a prospective rural Chinese cohort of 3,632 adults without MetS at baseline reported higher incident MetS with occasional tea consumption (OR 1.284, 95% CI 1.050–1.570) and with one to two tea-drinking episodes/day (OR 1.376, 95% CI 1.030–1.760) over a median 4.66 years (28). This genuinely discordant evidence should temper generalized cardiometabolic claims.

### 4.4 Interpreting observational and genetic evidence

The observational signal is not equivalent to causality. Even well-adjusted cohorts may retain confounding by socioeconomic status, smoking, alcohol use, physical activity, body weight, dietary quality, healthcare access, and other behaviors. Reverse causation is also possible if participants with illness reduce tea or caffeine intake. Tea can also substitute for sugar-sweetened drinks or alcohol; therefore, part of an apparent benefit may reflect displacement of less healthy beverages rather than an intrinsic effect of tea. The null Mendelian randomization findings in ref. 23 reduce confidence in a direct causal interpretation and are especially relevant to the review as a whole rather than to AF alone, although Mendelian randomization also depends on instrument strength, valid assumptions, and the genetic proxy used for tea intake. The most defensible interpretation is that observational associations are compatible with, but do not prove, cardiovascular benefit.

## 5 Exploratory subgroup findings and potential modifiers

Sex, age, comorbidity, and genetic background may contribute to heterogeneity, but most reported differences arise from subgroup analyses rather than prespecified formal interaction tests and should be considered exploratory. Some studies have reported sex- or age-related differences, while the JACC analysis suggested sex-specific patterns among MI survivors (21). Such findings require replication with formal interaction testing before they can guide individualized intake. Genetic variation affecting caffeine metabolism, including CYP1A2-related pathways, is biologically plausible, but the UK Biobank mortality associations did not materially differ by a caffeine-metabolism genetic score (20). Thus, patient heterogeneity is relevant to future precision-oriented research, but no clinically actionable demographic or genetic modifier of tea-related cardiovascular effects is currently established.

## 6 Dose-response evidence and exposure heterogeneity

Dose-response meta-analyses of prospective studies have generally reported lower cardiovascular mortality at moderate-to-higher tea intake (29, 30), and a meta-analysis in individuals with diabetes also reported inverse associations between tea intake and cardiovascular mortality (31). However, an optimal therapeutic dose cannot be inferred. Chung et al. standardized one cup to approximately 236 mL for their dose-response analysis (29), whereas primary studies used different cup volumes, frequencies, tea strengths, brewing temperatures and durations, and tea types. Tea chemistry is itself influenced by plant material and preparation conditions (32, 33). These differences limit direct conversion of epidemiological categories into a universal number of cups per day. Current evidence therefore supports describing dose-response associations rather than prescribing a standardized intake target.

## 7 Individualized tea selection: mechanistic rationale, clinical considerations, and evidence gaps

The six major types of tea (green, white, yellow, oolong, black, and dark tea) differ substantially in their bioactive profiles because of processing and fermentation (32). Non-fermented or minimally fermented teas, particularly green tea, retain relatively high concentrations of catechins such as EGCG; oxidation during black-tea processing promotes the formation of TFs and TRs; and post-fermented dark teas contain relatively greater amounts of TBs and fermentation-related products. These compositional differences provide a biological rationale for asking whether cardiovascular responses could vary by tea type and patient phenotype. However, no adequately powered head-to-head clinical trials have established that green, black, or dark tea is superior for coronary artery disease, AF, heart failure, MetS, or other cardiovascular conditions. Therefore, the original disease-directed concept of green tea for coronary disease, black tea for MI or stroke survivors, or dark tea for heart failure with metabolic dysfunction cannot currently be presented as evidence-based clinical guidance.

A more defensible personalized framework is to distinguish mechanistic hypotheses from practical patient-level considerations. Tea composition may define the biological hypothesis, whereas caffeine sensitivity, iron status, concomitant medications, overall dietary pattern, and, in selected patients with heart failure, total fluid intake may influence the suitability and amount of tea consumed. In this sense, individualized tea selection remains a translational research concept: future trials should test whether defined tea types or constituent profiles interact with cardiovascular phenotype, sex, age, caffeine metabolism, or gut-microbiome features to produce clinically meaningful differences in benefit or risk. Until such comparative evidence is available, personalization should emphasize tolerance and safety rather than presumed disease-specific efficacy.

## 8 Safety and potential interactions

Brewed tea and concentrated extracts should be distinguished when considering safety, and these considerations are central to any individualized framework. Traditional green-tea infusions are generally considered safe at customary intakes, whereas the European Food Safety Authority identified liver-enzyme elevations in intervention studies using green-tea supplements providing at least 800 mg EGCG/day (34). Tea taken with meals can reduce non-heme iron absorption (35), which is clinically relevant when iron deficiency is present or likely. Green-tea extract also reduced atorvastatin exposure in a small randomized crossover pharmacokinetic study, consistent with transporter-mediated interactions (36). A single case report described reduced warfarin anticoagulation during exceptionally high green-tea intake (37); this low-level evidence supports consistency of intake and clinical monitoring rather than blanket avoidance.

Caffeine should also be considered, especially in patients who attribute palpitations to caffeinated beverages. Contemporary evidence does not support assuming that ordinary caffeinated tea increases AF risk, and the 2026 American Heart Association scientific statement emphasizes heterogeneous individual responses and differences among caffeine sources; evidence from high-dose purified caffeine should not be extrapolated directly to ordinary tea (38). In selected patients with heart failure who are advised to restrict fluids, tea contributes to total fluid intake independently of its bioactive constituents. Finally, high-fluoride brick-tea exposure is a distinct regional safety issue: a survey across Tibet documented substantial dental and skeletal fluorosis in populations with heavy brick-tea consumption (39). These findings should not be generalized to all tea but are important when discussing dark or brick tea in high-exposure populations.

## 9 Challenges and future directions

Several gaps prevent clinical prescription of tea for cardiovascular prevention or prognosis. First, no adequately powered randomized trial of brewed tea with major cardiovascular events or cardiovascular mortality as a primary endpoint was identified in this review; most intervention trials evaluate surrogate risk factors. Second, exposure standardization remains difficult because cultivar, processing, dose, cup volume, brewing conditions, additives, and co-consumed foods alter chemical exposure. Third, future studies should measure relevant metabolites and account for bioavailability instead of extrapolating directly from high-concentration cell experiments. Fourth, causal inference requires better control of lifestyle confounding, beverage substitution, and reverse causation, with triangulation across randomized, genetic, and observational designs. Finally, the translational question raised by differences among tea types should be tested directly: prospective comparative trials could evaluate whether predefined cardiovascular phenotypes or patient characteristics modify responses to standardized tea interventions. Contemporary ESC prevention guidance and 2026 American Heart Association dietary guidance emphasize overall heart-healthy dietary patterns rather than disease-specific therapeutic tea prescriptions (40, 41). Thus, individualized tea selection is a promising research direction, but current clinical translation should prioritize evidence level, exposure context, patient preference, and safety.

## 10 Discussion

Current evidence supports biologically plausible cardiovascular effects of tea and recurring epidemiological associations with favorable cardiovascular outcomes, but causality remains uncertain because clinical evidence is dominated by observational studies and surrogate endpoints and remains susceptible to residual confounding and reverse causation. The lack of adequately powered randomized trials with hard cardiovascular endpoints is a major evidence gap. A methodological limitation of this narrative mini review is that the literature search was restricted to PubMed rather than conducted across multiple bibliographic databases; therefore, relevant studies indexed exclusively in other databases may have been missed. Accordingly, this review should be interpreted as a narrative synthesis rather than a systematic review.

Differences in tea composition and patient characteristics provide a rationale for individualized tea-consumption strategies, but such personalization should currently be regarded as a hypothesis-generating precision-nutrition framework rather than a disease-specific recommendation. Future studies should standardize tea exposure, directly compare tea types, incorporate bioavailability and relevant patient-level modifiers, and evaluate clinical cardiovascular endpoints. Until then, no specific tea type or dose should be prescribed or used in place of established preventive or therapeutic strategies.

## References

[1] Roth GA Mensah GA Johnson CO Addolorato G Ammirati E Baddour LM. Global burden of cardiovascular diseases and risk factors, 1990–2019: update from the GBD 2019 study. J Am Coll Cardiol. (2020) 76(25):2982–3021. 10.1016/j.jacc.2020.11.010 33309175 PMC7755038

[2] Tang GY Meng X Gan RY Zhao CN Liu Q Feng YB. Health functions and related molecular mechanisms of tea components: an update review. Int J Mol Sci. (2019) 20(24):6196. 10.3390/ijms20246196 31817990 PMC6941079

[3] Münzel T Camici GG Maack C Bonetti NR Fuster V Kovacic JC. Impact of oxidative stress on the heart and vasculature: part 2 of a 3-part series. J Am Coll Cardiol. (2017) 70(2):212–29. 10.1016/j.jacc.2017.05.035 28683969 PMC5663297

[4] Truong VL Jeong WS. Cellular defensive mechanisms of tea polyphenols: structure-activity relationship. Int J Mol Sci. (2021) 22(17):9109. 10.3390/ijms22179109 34502017 PMC8430757

[5] Ou HC Song TY Yeh YC Huang CY Yang SF Chiu TH. EGCG Protects against oxidized LDL-induced endothelial dysfunction by inhibiting LOX-1-mediated signaling. J Appl Physiol (1985). (2010) 108(6):1745–56. 10.1152/japplphysiol.00879.2009 20203069

[6] Guo BC Wei J Su KH Chiang AN Zhao JF Chen HY. Transient receptor potential vanilloid type 1 is vital for (-)-epigallocatechin-3-gallate mediated activation of endothelial nitric oxide synthase. Mol Nutr Food Res. (2015) 59(4):646–57. 10.1002/mnfr.201400699 25581901

[7] Ras RT Zock PL Draijer R. Tea consumption enhances endothelial-dependent vasodilation; a meta-analysis. PLoS One. (2011) 6(3):e16974. 10.1371/journal.pone.0016974 21394199 PMC3048861

[8] Ras RT Streppel MT Draijer R Zock PL. Flow-mediated dilation and cardiovascular risk prediction: a systematic review with meta-analysis. Int J Cardiol. (2013) 168(1):344–51. 10.1016/j.ijcard.2012.09.047 23041097

[9] Peng X Zhou R Wang B Yu X Yang X Liu K. Effect of green tea consumption on blood pressure: a meta-analysis of 13 randomized controlled trials. Sci Rep. (2014) 4:6251. 10.1038/srep06251 25176280 PMC4150247

[10] Zhang G Pan Y Cheng H Gong S Chu Q Chen P. Theaflavin: a natural candidate to restrain thrombosis. Food Funct. (2022) 13(14):7572–81. 10.1039/d2fo00152g 35815842

[11] Jin Y-R Im J-H Park E-S Cho M-R Han X-H Lee J-J. Antiplatelet activity of epigallocatechin gallate is mediated by the inhibition of PLCgamma2 phosphorylation, elevation of PGD2 production, and maintaining calcium-ATPase activity. J Cardiovasc Pharmacol. (2008) 51(1):45–54. 10.1097/FJC.0b013e31815ab4b6 18209568

[12] Joo HJ Park JY Hong SJ Kim KA Lee SH Cho JY. Anti-platelet effects of epigallocatechin-3-gallate in addition to the concomitant aspirin, clopidogrel or ticagrelor treatment. Korean J Intern Med. (2018) 33(3):522–31. 10.3904/kjim.2016.228 29050464 PMC5943656

[13] Chen K Chen W Liu SL Wu TS Yu KF Qi J. Epigallocatechingallate attenuates myocardial injury in a mouse model of heart failure through TGF-*β*1/Smad3 signaling pathway. Mol Med Rep. (2018) 17(6):7652–60. 10.3892/mmr.2018.8825 29620209 PMC5983962

[14] Wang S Wang H. Theaflavin-3,3'-digallate protects against myocardial ischemia/reperfusion injury and hypoxia/reoxygenation injury by activating the PI3 K/akt/mTOR pathway. J Mol Histol. (2025) 56(4):207. 10.1007/s10735-025-10453-z 40576913

[15] Heianza Y Ma W Manson JE Rexrode KM Qi L. Gut Microbiota metabolites and risk of Major adverse cardiovascular disease events and death: a systematic review and meta-analysis of prospective studies. J Am Heart Assoc. (2017) 6(7):e004947. 10.1161/JAHA.116.004947 28663251 PMC5586261

[16] Huang F Zheng X Ma X Jiang R Zhou W Zhou S. Theabrownin from pu-erh tea attenuates hypercholesterolemia via modulation of gut microbiota and bile acid metabolism. Nat Commun. (2019) 10(1):4971. 10.1038/s41467-019-12896-x 31672964 PMC6823360

[17] Zhang C Li M Li L Wan C Xia Y Ma H. Theabrownins from dark tea: formation, gut microbiota-mediated biotransformation, health benefits, and potential in functional food applications. Food Funct. (2025) 16(16):6346–68. 10.1039/D5FO02046H 40748185

[18] Lee MJ Maliakal P Chen L Meng X Bondoc FY Prabhu S. Pharmacokinetics of tea catechins after ingestion of green tea and (-)-epigallocatechin-3-gallate by humans: formation of different metabolites and individual variability. Cancer Epidemiol Biomarkers Prev. (2002) 11(10 Pt 1):1025–32. 12376503

[19] Wang X Liu F Li J Yang X Chen J Cao J. Tea consumption and the risk of atherosclerotic cardiovascular disease and all-cause mortality: the China-PAR project. Eur J Prev Cardiol. (2020) 27(18):1956–63. 10.1177/2047487319894685 31914807

[20] Inoue-Choi M Ramirez Y Cornelis MC Berrington de González A Freedman ND Loftfield E. Tea consumption and all-cause and cause-specific mortality in the UK biobank: a prospective cohort study. Ann Intern Med. (2022) 175(9):1201–11. 10.7326/M22-0041 36037472 PMC10623338

[21] Teramoto M Muraki I Yamagishi K Tamakoshi A Iso H. Green tea and coffee consumption and all-cause mortality among persons with and without stroke or myocardial infarction. Stroke. (2021) 52(3):957–65. 10.1161/STROKEAHA.120.032273 33535784 PMC7903984

[22] Liu DC Yan JJ Wang YN Wang ZM Xie ZY Ma Y. Low-dose green tea intake reduces incidence of atrial fibrillation in a Chinese population. Oncotarget. (2016) 7(51):85592–602. 10.18632/oncotarget.12243 27683043 PMC5356761

[23] Cai D Chen J Wu Y Jiang C. No causal association between tea consumption and 7 cardiovascular disorders: a two-sample Mendelian randomization study. Front Genet. (2022) 13:989772. 10.3389/fgene.2022.989772 36531229 PMC9748479

[24] Mottillo S Filion KB Genest J Joseph L Pilote L Poirier P. The metabolic syndrome and cardiovascular risk a systematic review and meta-analysis. J Am Coll Cardiol. (2010) 56(14):1113–32. 10.1016/j.jacc.2010.05.034 20863953

[25] Liu W Wan C Huang Y Li M. Effects of tea consumption on metabolic syndrome: a systematic review and meta-analysis of randomized clinical trials. Phytother Res. (2020) 34(11):2857–66. 10.1002/ptr.6731 32578328

[26] Ghoflchi S Mohammadi H Teimouri M Imani M Hosseini H. The association between green tea intake and metabolic syndrome: a systematic review and meta-analysis of randomized controlled trials. Clin Ther. (2025) 47(12):e1–9. 10.1016/j.clinthera.2025.09.011 41073208

[27] Yang Y Yu D Piao W Huang K Zhao L. Association between habitual tea consumption and metabolic syndrome and its components among Chinese adults aged 18∼59 years: based on China nutrition and health surveillance 2015–2017. Nutrients. (2022) 14(17):3502. 10.3390/nu14173502 36079760 PMC9459911

[28] Yu S Wang B Li G Guo X Yang H Sun Y. Habitual tea consumption increases the incidence of metabolic syndrome in middle-aged and older individuals. Nutrients. (2023) 15(6):1448. 10.3390/nu15061448 36986178 PMC10055940

[29] Chung M Zhao N Wang D Shams-White M Karlsen M Cassidy A. Dose-Response relation between tea consumption and risk of cardiovascular disease and all-cause mortality: a systematic review and meta-analysis of population-based studies. Adv Nutr. (2020) 11(4):790–814. 10.1093/advances/nmaa010 32073596 PMC7360449

[30] Kim Y Je Y. Tea consumption and risk of all-cause, cardiovascular disease, and cancer mortality: a meta-analysis of thirty-eight prospective cohort data sets. Epidemiol Health. (2024) 46:e2024056. 10.4178/epih.e2024056 38938012 PMC11573487

[31] Ding L Wang HP Zhao JY Zhao X Sha Y Qin LQ. Coffee and tea consumption and cardiovascular disease and all-cause and cause-specific mortality in individuals with diabetes mellitus: a meta-analysis of prospective observational studies. Front Nutr. (2025) 12:1570644. 10.3389/fnut.2025.1570644 40525136 PMC12168468

[32] Wong M Sirisena S Ng K. Phytochemical profile of differently processed tea: a review. J Food Sci. (2022) 87(5):1925–42. 10.1111/1750-3841.16137 35368105

[33] Lin YS Tsai YJ Tsay JS Lin JK. Factors affecting the levels of tea polyphenols and caffeine in tea leaves. J Agric Food Chem. (2003) 51(7):1864–73. 10.1021/jf021066b 12643643

[34] FSA Panel on Food Additives and Nutrient Sources added to Food (ANS), Younes M Aggett P Aguilar F Crebelli R Dusemund B. Scientific opinion on the safety of green tea catechins. EFSA J. (2018) 16(4):e05239. 10.2903/j.efsa.2018.5239 32625874 PMC7009618

[35] Disler PB Lynch SR Charlton RW Torrance JD Bothwell TH Walker RB. The effect of tea on iron absorption. Gut. (1975) 16(3):193–200. 10.1136/gut.16.3.193 1168162 PMC1410962

[36] Abdelkawy KS Abdelaziz RM Abdelmageed AM Donia AM El-Khodary NM. Effects of green tea extract on atorvastatin pharmacokinetics in healthy volunteers. Eur J Drug Metab Pharmacokinet. (2020) 45(3):351–60. 10.1007/s13318-020-00608-6 31997084

[37] Taylor JR Wilt VM. Probable antagonism of warfarin by green tea. Ann Pharmacother. (1999) 33(4):426–8. 10.1345/aph.18238 10332534

[38] Marcus GM Hu FB Van Dam RM Cornelis MC Dewland TA Kang JH. Caffeine and cardiovascular disease: a scientific statement from the American Heart Association. Circulation. (2026) 154. Online ahead of print. 10.1161/CIR.0000000000001454 42473796

[39] Fan Z Gao Y Wang W Gong H Guo M Zhao S. Prevalence of brick tea-type fluorosis in the Tibet autonomous region. J Epidemiol. (2016) 26(2):57–63. 10.2188/jea.JE20150037 26499132 PMC4728115

[40] Visseren FLJ Mach F Smulders YM Carballo D Koskinas KC Bäck M. 2021 ESC guidelines on cardiovascular disease prevention in clinical practice. Eur Heart J. (2021) 42(34):3227–337. 10.1093/eurheartj/ehab484 34458905

[41] Lichtenstein AH Khera A Anderson CAM Appel LJ DeSilva DM Gardner C. 2026 Dietary guidance to improve cardiovascular health: a scientific statement from the American Heart Association. Circulation. (2026) 153(18):e1285–95. 10.1161/CIR.0000000000001435 41914202

